# Endophytic fungi of *Panax sokpayensis* produce bioactive ginsenoside Compound K in flask fermentation

**DOI:** 10.1038/s41598-024-56441-3

**Published:** 2024-04-23

**Authors:** Subecha Rai, Laishram Shantikumar Singh, Ramanan Uma Shaanker, Kumaraswamy Jeyaram, Tithi Parija, Dinabandhu Sahoo

**Affiliations:** 1 Institute of Bioresources and Sustainable Development (IBSD), Sikkim Centre, DBT, Tadong, Gangtok, Sikkim 737102 India; 2 School of Biotechnology, KIIT-Deemed to be University, Campus XI, Patia, Bhubaneshwar, Odisha 751024 India; 3 Department of Microbiology, Assam Down Town University, Guwahati, Assam 781026 India; 4 School of Ecology and Conservation, Department of Crop Physiology, University of Agricultural Sciences, GKVK, Bellary Road, Bangalore, Karnataka 560065 India; 5 Department of Botany, University of Delhi, Delhi, 110007 India

**Keywords:** *Panax sokpayensis*, Endophytes, Fungi, Ginsenoside, Compound K, Biotechnology, Chemical biology, Microbiology

## Abstract

Endophytes of *Panax* have the potential to produce their host plant secondary metabolites, ginsenosides. *Panax sokpayensis,* an endemic traditional medicinal plant of the Sikkim Himalayas was explored for the isolation of endophytic fungi. In the present study, we have isolated 35 endophytic fungal cultures from the rhizome of *P. sokpayensis* and screened for ginsenosides production by HPLC by comparing the peak retention time with that of standard ginsenosides. The HPLC analysis revealed that out of 35 isolates, the mycelial extracts of four fungal endophytes (PSRF52, PSRF53, PSRF49 and PSRF58) exhibited peaks with a similar retention time of the standard ginsenoside, Compound K (CK). LC–ESI–MS/MS analysis led to the confirmation of ginsenoside CK production by the four fungal endophytes which showed a compound with m/z 639.6278, similar to that of standard ginsenoside CK with yield in potato dextrose broth flask fermentation ranging from 0.0019 to 0.0386 mg/g of mycelial mass in dry weight basis. The four prospective fungal endophyte isolates were identified as *Thermothielavioides terrestris* PSRF52, *Aspergillus* sp. PSRF49, *Rutstroemiaceae* sp. strain PSRF53, and *Phaeosphaeriaceae* sp. strain PSRF58 based on ITS sequencing. The present finding highlights the need for further study on growth optimization and other culture parameters to exploit the endophytes as an alternative source for ginsenoside CK production.

## Introduction

The *Panax* genus of the *Araliaceae* family is a well-known medicinal plant with its earliest records found in Traditional Chinese Medicine (TCM) literature. TCM reports the use of *Panax* rhizome extract as a health supplement and the leaves for preparing tea and other concoctions, which are used mainly as a tonic to invigorate weak bodies^1,2^. With the advent of time, several studies have attributed the ginsenosides present in *Panax* rhizome to their medicinal properties^3^. Its acceptance by the US Pharmacopoeia in 1840 drastically increased the use of *Panax* rhizome as a medicine worldwide, particularly in the Western World. As *Panax* grows very slowly and requires 5–7 years to produce a reasonable size of rhizome, it requires specific geographical conditions for its growth, like high elevation and temperate climate of the Himalayan region^1,4^, and primarily collected from the wild, making the *Panax* rhizome an expensive commodity^1,2^. Although the Chinese side of the Himalayan region is well-explored for *Panax* diversity and its medicinal properties, very few studies have investigated the Indian side of the Himalayas.

The ginsenosides are a group of triterpene saponins excessively produced in the *Panax* rhizome and are known to have multifaceted pharmacological functions such as anti-ageing, analgesic, antidiabetic, antipyretic, anticarcinogenic, antistress, and antifatigue^5–8^. The main ginsenosides present in *Panax* rhizome such as ginsenosides Rg1, Rd, Rc, Rb1 and Rb2, contain multi-sugar moieties, exhibit little pharmacological activities, and are not readily absorbed by the human body^9^. The deglycosylation of these inactive ginsenosides into active ginsenosides by intestinal bacteria and digestive enzymes allows their absorption by the human digestive system. Ginsenoside Compound K (CK), a rare protopanaxadiol type of ginsenoside, is known to be the major contributor to the activity of ginsenosides and has been shown to possess anti-inflammation, hepatoprotection, anti-diabetes and anti-cancer activity^10–13^. CK is currently synthesized by enzymatic or microbial deglycosylation of the main protopanaxadiol category of ginsenosides (Rb1, Rb2, Rd and Rc)^9^. However, its manufacturing is limited due to the availability of raw materials, as the *Panax* plant and the quality rhizome require a long time for cultivation^14^.

Since the discovery of the anti-cancer compound “taxol”, the ability of endophytes to produce bioactive metabolites in medicinal plants has gained attraction^15^. Very few studies have reported ginsenoside production by the *Panax* endophytes. The fungal endophytes of *Panax ginseng*, *Fusarium* sp*.* Pg27 and *Aspergillus* sp. Pg30 could produce ginsenoside Rb2 and *Verticillium* sp. Pg42-1 could produce ginsenoside Rc^16^. A similar study, reported in vitro production of rare ginsenosides Rg3 and Rh2 by endophytic bacteria of *P. ginseng, Agrobacterium rhizogenes* PDA-2^17^. In this context, screening of ginsenoside-producing endophytes of *Panax* rhizome is gaining momentum as an alternative sustainable method for industrial ginsenoside production.

Among all the *Panax* species found worldwide, *Panax ginseng*, *Panax quinquefolius* and *Panax notoginseng* are the most studied for their ginsenosides and therapeutic applications^18^. *Panax sokpayensis* is endemic to Sikkim (an Indian part of the Himalayan region) and faces a threat due to excessive harvesting from the wild and illegal marketing^19–21^. An earlier study by Gurung et al., showed the major ginsenoside content in *P. sokpayensis* which is at par with that of *P. ginseng* and *P. notoginseng*^20^. In the present study, we have screened the fungal endophytes of *P. sokpayensis* for their ability to produce ginsenosides under in vitro conditions.

## Materials and methods

### Sampling

**Figure 1:**
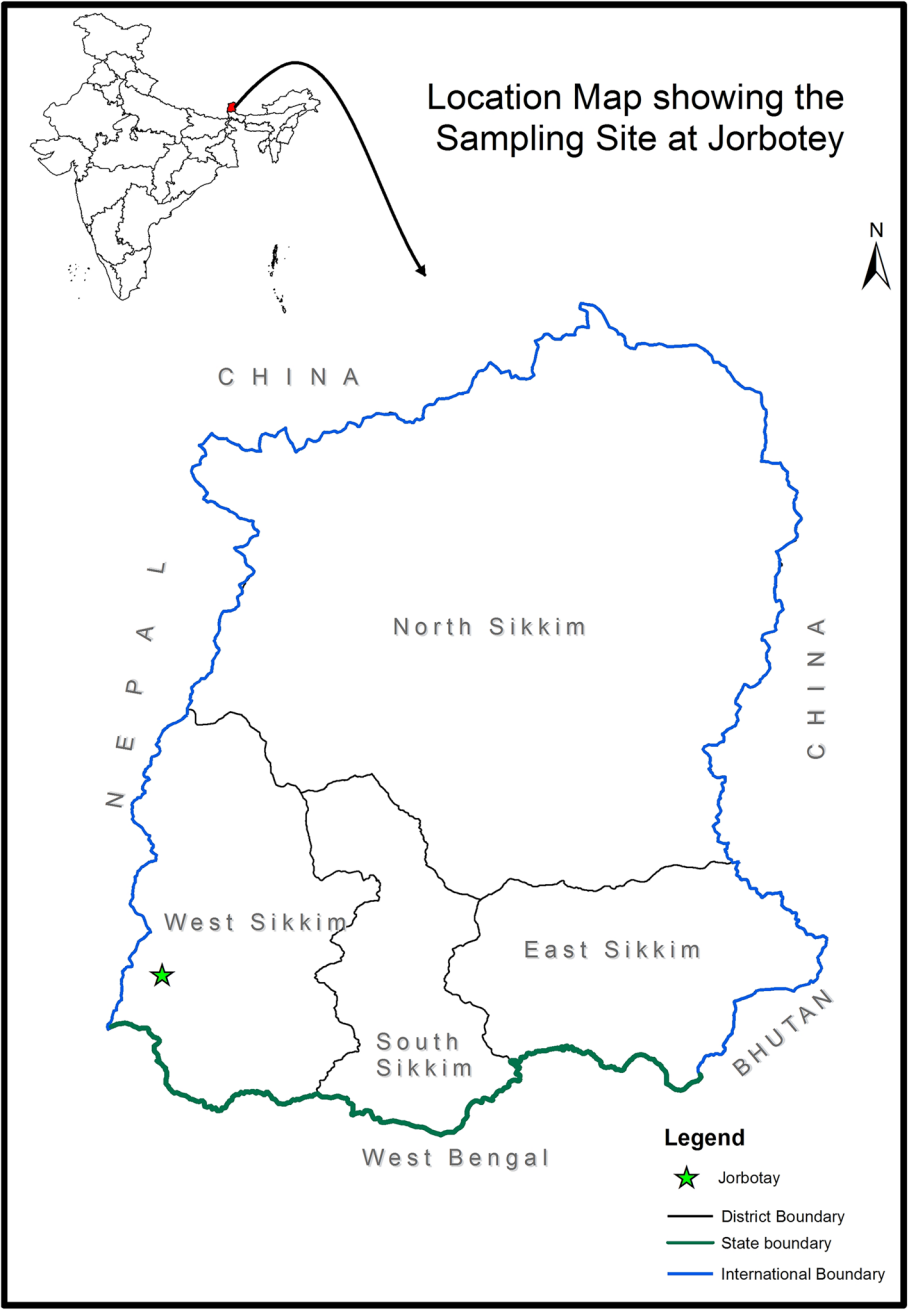
Location map showing the sampling site at Jorbotay, Sikkim, India. The green colour star indicates the sample collection site (Map was created using the software ArcGIS 9.3 and 10.8.1; https://arcgis.software.informer.com/9.3/).

Five rhizome samples of *P. sokpayensis* were collected from Jorbotay (Latitude: 27°16′54.84″ N; Longitude: 88°5′8.12″E; Altitude: 2297 m), Uttarey, Gyalshing district of Sikkim, India (Fig. 1). The samples were collected in a sterile plastic bag and transported to the laboratory in a cooler box, and endophyte isolation was performed within 24 h of collection. The sample collection was carried out according to the guideline of the Sikkim State Government after obtaining the research permit (vide letter no.: 78/GOS/FEWMD/BD-R-2015/CCF(T&HQ)35 dated May 15, 2017) from the office of the Chief Conservator of Forest (T&HQ) cum CWLW, Department of Forest, Environment and Wildlife Management, Government of Sikkim, Deorali, Gangtok–737102, Sikkim. The plant specimen was authenticated by the Botanical Survey of India, Sikkim Himalayan Regional Centre, Gangtok, 737103, Sikkim, Ministry of Environment, Forest and Climate Change, Government of India (vide letter no. SHRC/5/40/2021-Tech-273) and the sample number accorded was IBSD-SC-M11.

### Isolation of fungal endophytes from rhizome of* P. sokpayensis*

**Figure 2:**
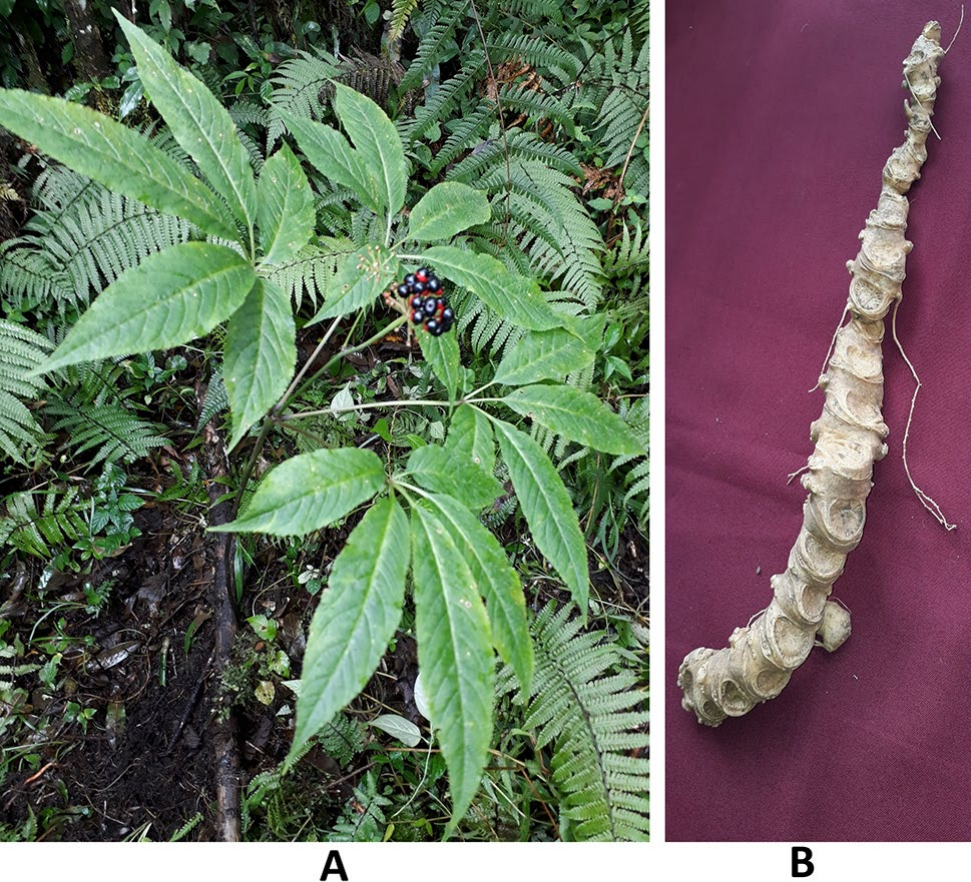
(**A**) *Panax sokpayensis* Shiva K*.* Sharma & Pandit in natural habitat and (**B**) rhizome of the plant.

With a few minor modifications, the methodology outlined by Mazumder et al. was adopted to isolate endophytic fungi from the rhizome of *P. sokpayensis*^22^. The collected rhizome sample (Fig. 2) was rinsed meticulously under running tap water to eliminate adhered soil and dust particles which was followed by washing with sterile distilled water and cut into small pieces (3–4 cm) under sterile conditions. The rhizome fragment was then subjected to surface sterilization under laminar airflow. Surface sterilization is a crucial step to remove any epiphytic microorganism which was carried out by following the standard protocol; the rhizome fragment was first dipped in 70% (v/v) ethanol for 5 min, followed by treatment with sodium hypochlorite (6%) for 5 min and then with 70% (v/v) ethanol for 30 s. Finally, the sterilized rhizome fragment was rinsed with sterile distilled water. The outer tissues were removed using a sterile scalpel after drying the sample aseptically. The sterilized rhizome fragment was cut into small pieces (0.5 × 0.5 cm) and placed on potato dextrose agar (PDA) medium (HiMedia Laboratories Pvt. Ltd, Mumbai, India) amended with streptomycin (50 μg/ml) in Perti plates and incubated at 25 ± 1 °C for 30 days. Following incubation, plates were checked and hyphal tips of fungi, emerging out of the plated rhizome pieces, were picked and grown on PDA medium. The endophytic fungal isolates were purified by hyphal tip culturing, and the isolates were preserved as glycerol stock in cryovials at − 80 °C. As an additional test of surface sterilization, aliquots (100 µl) of the final rinse water of rhizome fragment were also plated onto a PDA medium. No growth on the PDA plate inoculated with final rinse water ensured the efficacy of the surface sterilization process and absence of any epiphytic contamination.

### Extraction of ginsenosides from endophytic fungal culture

The pure culture of each fungal endophyte isolate was cultured in potato dextrose broth (PDB) (HiMedia Laboratories Pvt. Ltd, Mumbai, India). Fungal hyphae from freshly grown culture were inoculated into 150 ml sterilized PDB contained in 250 ml Erlenmeyer flask and incubated at 25 ± 1 °C for ten days without shaking. After incubation, culture supernatant and fungal mycelia were separated by centrifugation at 10,000 rpm for 10 min (Sorvall Biofuge Primo) for the extraction of ginsenosides. The fungal mycelia were rinsed with sterile distilled water, dried in an oven at 45 °C for two days and then crushed into powder using liquid nitrogen. One gram of the mycelial powder was extracted using 20 ml methanol by subjecting it to ultrasonication at 30 °C for 30 min. The extracted mixture was filtered using Whatman’s filter paper and the resultant filtrate was concentrated in a rotary evaporator (BUCHI). The methanol extract obtained for each isolate was then transferred to a 2 ml vial and was subjected to drying using a vacuum concentrator (SpeedVac Concentrator, ThermoFisher Scientific) and stored at − 20 °C for further analysis^23^. The culture supernatant of 20 ml was extracted with an equal volume (20 ml) of ethyl acetate, concentrated using a rotary evaporator (BUCHI) and subjected to drying as above for analysis. One ml of Methanol (HPLC grade) was added to the dried extracts and filtered using Whatman’s 0.3 μm syringe filter which was used for HPLC analysis.

### HPLC analysis

The production of ginsenosides by the fungal endophytes was analyzed by using reverse-phase HPLC (LC-20AT, Shimadzu) with Luna 5 μm C18 (250 × 4.6 mm) Column (Phenomenex), using Photodiode Array Detector (SPD-M 20A) with an oven temperature of 60 °C. The wavelength of the PAD detector was kept at 205 nm, and the flow rate was maintained at 1 ml/min with an injection volume of 20 μl. Acetonitrile (Pump A) and (Pump B) HPLC grade water in binary gradient mode was used as mobile phase in the ratios of the solvents A: B at 25% B held for 3 min; to 85% B in 12 min; 85% B held for 1 min respectively. The system then returned to the initial condition in 1 min after 100% of B. The reference ginsenosides viz. CK, Rb1, Rb2, Rb3, Rd, Re, Rf, Rg1, Rg2, Rg3 and Rh2 (ChromaDex, Irvine, USA) were used as standards during the analysis. The standard solutions of all reference ginsenosides were prepared using 100% methanol (HPLC grade) at 1 mg/ml concentration while the standard solution of Rg3 at 1 mg/ml concentration was prepared using dimethyl sulfoxide (DMSO) as per the manual provided by Chromadex. 20 μl each of the different standard solutions were injected into the HPLC system and run separately for 20 min. Ginsenosides' presence in the extract samples was analyzed by comparing the retention time with the standards by following the method of detection described by Sarang et al.^23^. The samples spiked with ginsenoside standard were run for confirmation^24,25^.

### LC–ESI–MS/MS detection of ginsenosides

The mycelial extracts of the fungal endophytes were further subjected to LC–ESI–MS/MS analysis for the presence of ginsenosides detected tentatively by the HPLC. The HPLC (Dionex Ultimate 3000, ThermoFisher Scientific) with C18 (250 × 4.6 mm) column (Syncromis), Acetonitrile and Water with a flow rate of 0.4 ml/min, and Impact HD mass spectrometer having ESI and Q-TOF (Bruker) was used to perform the mass spectral analyses. Nitrogen was used as both nebulizing gas and desolvation gas at flow rates of 3 bar and 12 L/min, respectively. The temperatures of both the electrospray source and desolvation gas were kept at 180 °C, and capillary potential was set at 3.0 kV. After selecting precursor ions by the first quadrupole mass analyzer, Collision RF was carried out in the range of 300–1500 vpp in the hexapole collision cell. The quadrupole ion energy used was 5 eV, and the collision cell energy used was 10 eV^24,25^. High-resolution mass spectra were acquired in both positive and negative-ion modes by scanning over the m/z range of 40–2000. The analyses of the mass of the ginsenosides were repeated twice for each fungal culture by comparing them with the ginsenoside standards.

### Molecular identification of the ginsenoside-producing fungal endophytes

Out of the 35 endophytic fungal isolates screened, four isolates capable of producing ginsenosides in in vitro conditions were identified by molecular method by ITS rDNA sequencing. The four endophytic fungal isolates viz. PSRF52, PSRF53, PSRF49 and PSRF58 were freshly cultured separately in 150 ml sterilized PDB in 250 ml Erlenmeyer flask and incubated for 10 days in a rotary shaker maintained at 25 ± 1 °C with 140 rpm. The fungal mycelia were separated by centrifugation at 10,000 rpm for 10 min (Sorvall Biofuge Primo), subjected to freeze-drying and DNA extraction was performed by adopting the Cetyltrimethylammonium bromide method as described by Vainio et al.^26^. The primer pair ITS1 (5′-TCCGTAGGTGAACCTGCGG-3′) and ITS4 (5′-TCCTCCGCTTATTGATATGC-3′) as per White et al.^27^ was used for ITS gene amplification using a thermal cycler (Bio-Rad) by following PCR parameters: denaturation at 94 °C for 3 min which was followed by 30 cycles of 94 °C for 15 s, 50 °C for 1 min, 72 °C for 45 s, and final extension at 72 °C for 7 min. The QIA quick PCR purification kit (Qiagen) was used to purify the PCR product, further sequenced using an ABI 3730xl Genetic Analyzer (Eurofins Genomics India Pvt. Ltd., Bangalore, India). The NCBI-BLAST search was carried out by using the rRNA/ITS database containing internal transcribed spacer region (ITS) from fungal type and reference materials sequences to obtain the relatedness of the endophytic fungal sequences^28^. The sequence having maximum query coverage, maximum identity score and highest homology was taken as a reference to assign the identity of the new fungal endophyte isolate. Observing the maximum identity score, the closest reference sequences were chosen and aligned using the multiple alignment software program Clustal W^29^. The Maximum Likelihood method^30^ based on the Kimura 2-parameter model was adopted for the construction of phylogenetic trees^31^, applying the Neighbor-Joining approach, and by taking bootstrap testing^32^ of 1000 replicates using MEGA6^33^. The ITS sequences of these four endophytic fungal isolates (PSRF52, PSRF53, PSFR49 and PSRF58) were deposited to NCBI GenBank and their accession numbers are ON963979, MN888777, MN888810 and MN888811 respectively.

## Results

### HPLC analysis evidences ginsenoside production by fungal endophytes of *P. sokpayensis*

**Figure 3:**
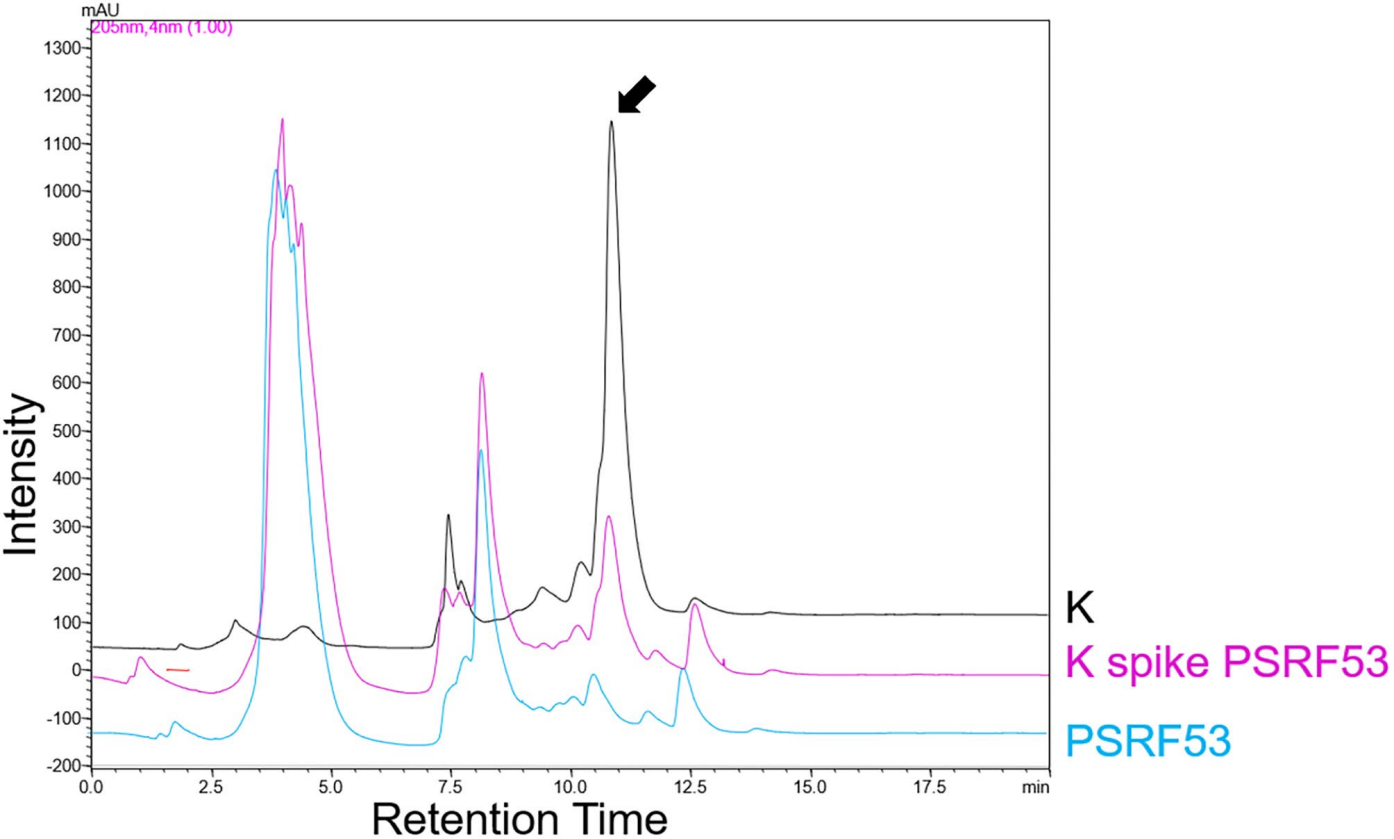
Overlay HPLC chromatogram of Ginsenoside Compound K peak with retention time of 10.8 min (denoted in black), Spiked samples: mycelial extract of endophytic isolate PSRF53+ Compound K (denoted in pink), and the mycelial extract of endophytic isolate PSRF53 (denoted in blue). The arrow indicates the peak of Ginsenoside Compound K.

We have screened 35 endophytic fungal isolates of *Panax sokpayensis* rhizome for ginsenosides production by HPLC by comparing the peak retention time with known ginsenosides standards. The HPLC analysis of the mycelial extract of six fungal endophytes exhibited peaks with a similar retention time of the ginsenosides Rg1 (7.8 min), CK (10.8 min), Rf (8.5 min) and Rg2 (8.4 min). The culture supernatant extract of the fungal isolates did not show peaks having an equal retention time as that of the ginsenoside standards analyzed. Further, we spiked the mycelial extracts with the ginsenoside standards Rg1, CK, Rf and Rg2 to validate the similarity in retention times of the detected ginsenoside compounds. Only four fungal endophytes (PSRF52, PSRF53, PSRF49 and PSRF58) showed overlapping single peaks of ginsenoside Rg1 (7.8 min), Rf (8.5 min) and CK (10.8 min) in the spiked HPLC run (Fig. 3), which were subjected to mass spectrometry for confirmation.

### LC–ESI–MS/MS analysis confirms ginsenoside-CK production by fungal endophytes

**Figure 4:**
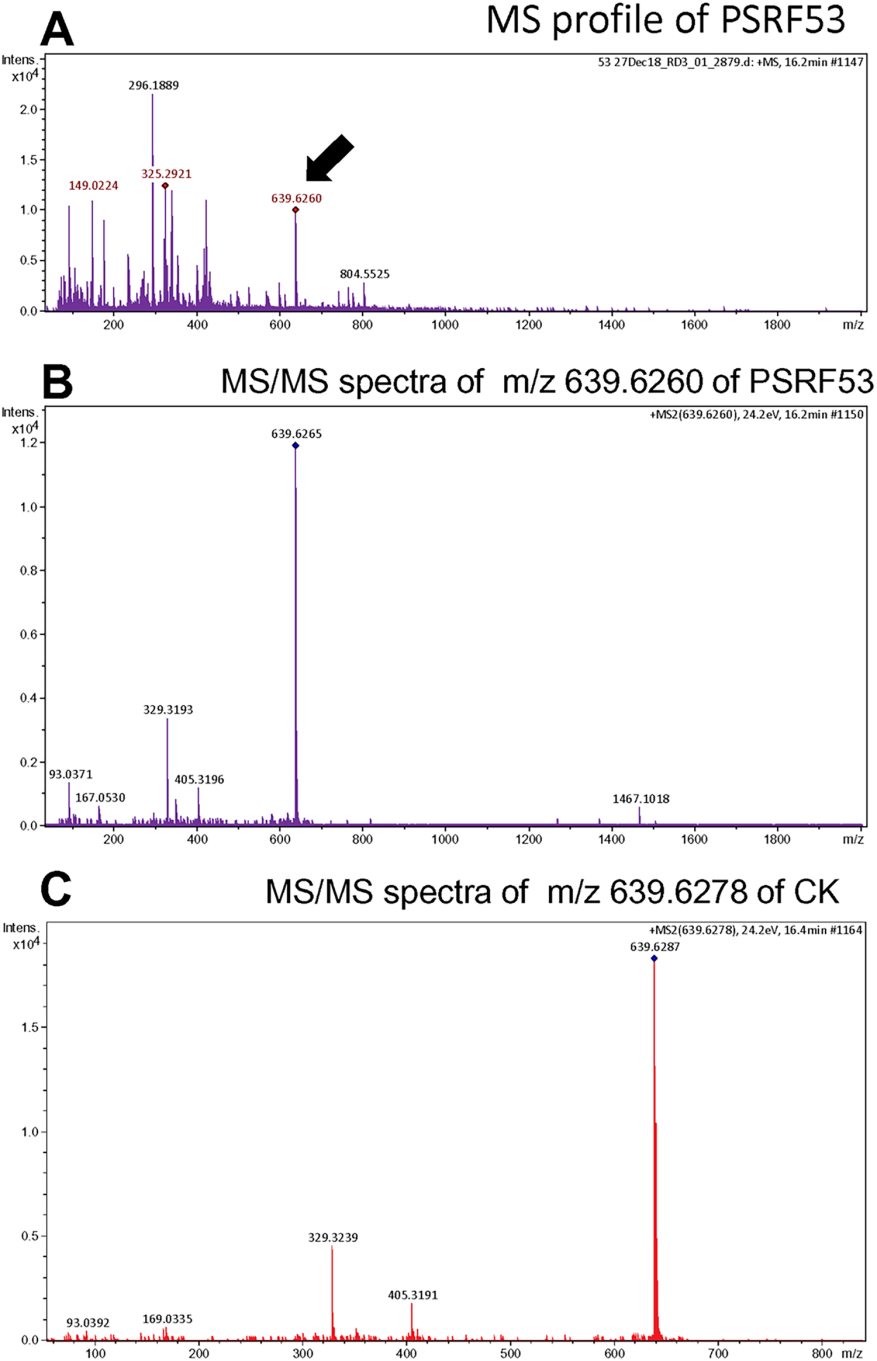
LC–ESI–MS/MS spectra. (**A**) The first mass spectra of mycelial extract of endophytic isolate PSRF53, the arrow indicates the molecular ion of ginsenoside Compound K (CK) at m/z 639.6260. (**B**) The MS/MS spectra of m/z 639.6260 of endophyte isolate PSRF53. (**C**) The MS/MS spectra of m/z 639.6278 of ginsenoside Compound K standard.

To confirm the ginsenosides detected by HPLC analysis, we subjected the mycelial extracts of the four endophytic fungi (PSRF52, PSRF53, PSRF49 and PSRF58) to LC–ESI–MS/MS analysis. The ginsenoside standards have a neutral mass of m/z 783.4885, 784.4885 and 621.4368 for Rf, Rg2 and CK respectively. The m/z profile of the mycelial extract of four fungal endophyte isolates (PSRF49, PSRF52, PSRF53 and PSRF58) did not coincide with those of ginsenoside standards Rf and Rg2. However, molecular ion peaks of CK[M + NH_4_]^+^ (m/z 639.6278) were observed (Fig. 4). The presence of CK was identified by comparing the retention time of 16.4 min and the most abundant ion of 639.6260 in the reference standard and the mycelial extracts of the endophytes. In MS/MS fragmentation, the ion first produced fragment ions at m/z 329.32, m/z 167 and m/z 93. This fragmentation pattern was identical to that of the standard CK. The concentration of ginsenoside CK production by the fungal cultures was calculated based on the LC–MS signal intensity of peak at the retention time of 16.4 min with m/z of 639.6260 in comparison to the known concentration of standard ginsenoside CK (ChromaDex, Irvine, USA). The ginsenoside CK production in PDB flask fermentation by the four endophytic fungal cultures was PSRF49 (0.0386 mg/g), PSRF52 (0.0028 mg/g), PSRF53 (0.0337 mg/g) and PSRF58 (0.0019 mg/g) of mycelial mass on a dry weight basis.

### Molecular identification of ginsenoside CK-producing fungal endophytes

**Figure 5:**
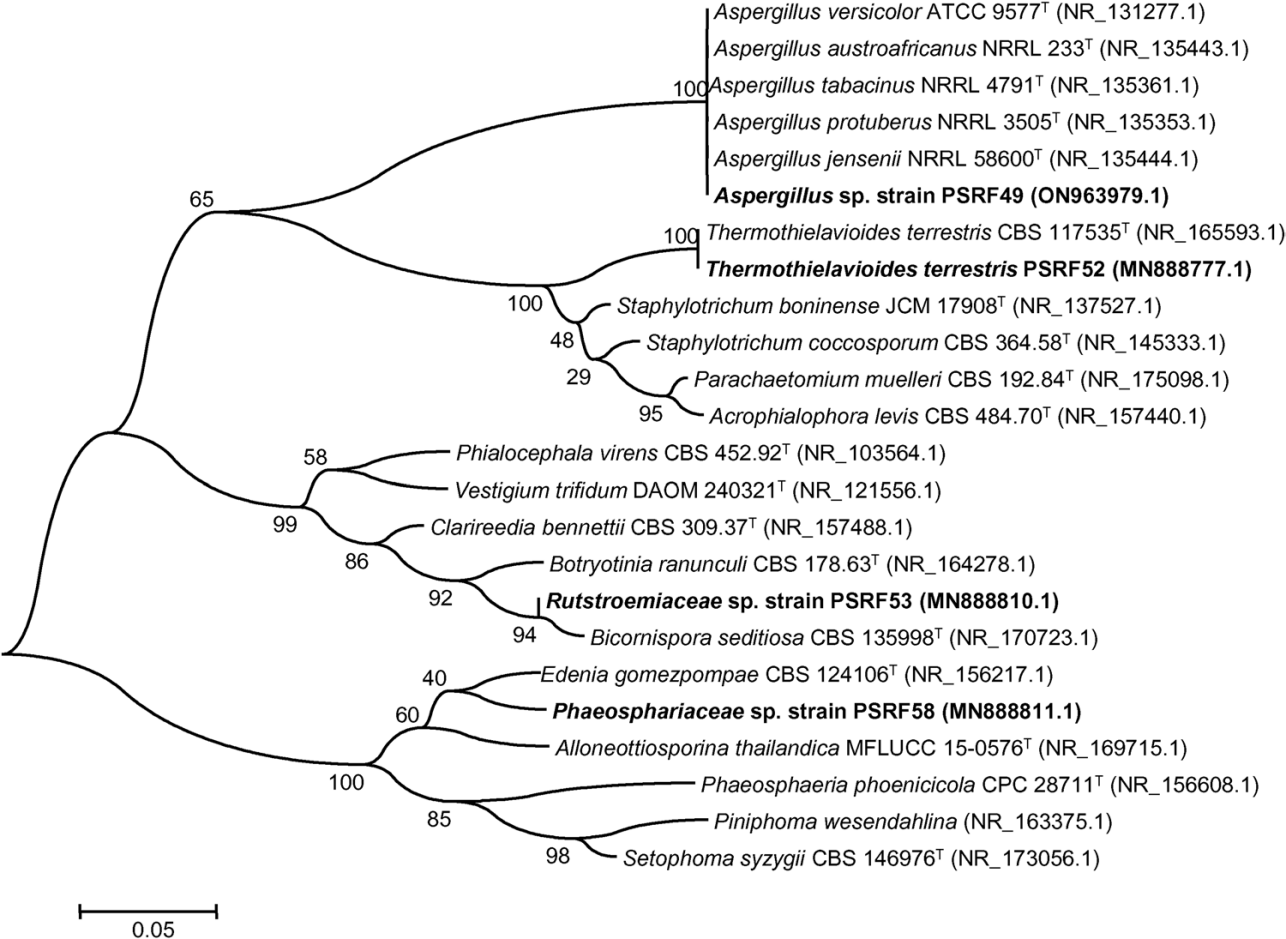
The phylogenetic tree based on ITS rDNA sequences constructed using the Maximum Likelihood method^30^ based on the Kimura 2-parameter model^31^ shows the taxonomic positions of four endophytic fungal isolates of *P. sokpayensis* produces ginsenoside Compound K in flask fermentation.

We used ITS amplicon sequencing to identify the four potential CK-producing fungal endophyte isolates (PSRF49, PSRF52, PSRF53, and PSRF58). The ITS sequence of the four fungal endophyte isolates were compared separately with the related reference sequences of type strains in the GenBank database. The sequence similarity analysis by NCBI-BLAST resulted in 99.64% sequence similarity for isolate PSRF52 with *Thermothielavioides terrestris* (CBS 117535), 99.63% sequence similarity for isolate PSRF49 with *Aspergillus austroafricanus* NRRL 233 (NR_135443.1) and 99.29% with *Aspergillus versicolor* ATCC 9577 (NR_131277.1). The isolate PSRF53 showed less than 96% similarity with *Bicornispora seditiosa* CBS 135998 of the family *Rutstroemiaceae* of potentially novel species. Similarly, the isolate PSRF58 showed less than 93% sequence similarity with *Edenia gomezpompae* CBS 124106 (NR_156217.1) and *Setophoma syzygii* CBS 146976 (NR_173056.1) of the family *Phaeosphaeriaceae*. As the isolate PSRF 53 and PSRF 58 showed novelty in their sequence with less than 96% sequence similarity with the available type strains reference sequences, their identity was limited at the family level. Thus, the four fungal endophyte isolates viz. PSRF52, PSRF49, PSRF53 and PSRF58 have been identified as *Thermothielavioides terrestris* PSRF52, *Aspergillus* sp. strain PSRF49, *Rutstroemiaceae* sp. strain PSRF53 and *Phaeosphaeriaceae* sp. strain PSRF58 respectively. The phylogenetic tree based on the Maximum Likelihood method shows the taxonomic position of these four endophytic fungal isolates in Fig. 5.

## Discussion

Studies on the endophytes of *Panax* spp. mostly focus on the diversity, biotransformation of ginsenosides and changes in the activity of ginsenosides^34–37^. These include fungi namely *Phoma*, *Fusarium, Trichoderma,* and *Setophoma* in *P. ginseng*^38^; *Trichoderma, Chaetomium, Fusarium, Aspergillus, Penicillium, Drechmeria, Emericella, Myrothecium* and *Preussia* in *P. notoginseng*^18,39–41^; and *Cladosporium* in *P. quinquefolius*^42^ with different bioactivities^43–45^. All these previous reports underscore the difference in the pattern of dominance of the fungal species, indicating the specificity of the distribution of endophytes depends on different geographical locations, environmental conditions as well as plant species^46^. Among these, only a few studies have been conducted on the ability of the fungal endophytes of *Panax* to produce ginsenosides. The fungal endophytes of *Panax ginseng*, particularly *Aspergillus* spp. cultures reported with triterpenoids saponins production up to 144 mg/L in the flask fermentation^16^. *Agrobacterium*, an endophytic bacterium of *P. ginseng* reported with ginsenosides Rg3, Rb1 and Rh2 production with a concentration ranging from 18 to 62 mg/L in the culture media^17^. The present study confirmed the ginsenoside CK production by four fungal endophytes of *P. sokpayensis* in PDB flask fermentation, with a maximum ginsenoside CK yield of 38.6 mg/kg in mycelial mass on a dry weight basis by *Aspergillus* sp. PSRF49. This is the first report of ginsenoside CK production by the fungal endophytes of *Panax* and the first investigation on endophytes of *Panax sokpayensis.*

Our study showed that four fungal endophytes of *Panax sokpayensis,* namely *Thermothielavioides terrestris* PSRF52, *Aspergillus* sp. strain PSRF49, *Rutstroemiaceae* sp. strain PSRF53 and *Phaeosphaeriaceae* sp. strain PSRF58 could produce the ginsenoside CK under in vitro conditions. *Thielavia terrestris* is known for producing industrially important enzymes and bioactive compounds known as thielavialides^46^. Until now, *Thielavia* has been reported as an endophyte only in the medicinal plant *Physalis alkekengi*^47^. The species of *Aspergillus*, particularly *Aspergillus versicolor,* as a fungal endophyte, has been found in several medicinal plants like *Anoectochilus roxburghii*, *Lycoris radiata, Euphorbia royleana, Pulicaria crispa* and *Avicennia marina,* and has been shown to possess the ability to synthesize many anti-microbial compounds and various pharmacologically important compounds^48^. The member *Rutstroemiaceae*, particularly *Lambertella corni-maris* was once reported as an endophyte in the leaves of *Abies koreana*^49^. However, the members of *Phaeosphaeriaceae,* particularly *Setophoma* spp. have been reported as an endophyte of *Panax ginseng* and several other plants^50–52^.

Among the ginsenosides discovered, CK is considered the main bioactive compound that brings about the various therapeutic activities of ginsenosides^12,53,54^. CK is a protopanaxadiol type of ginsenoside that has gained importance due to its high permeability leading to neuroprotective, immune-modulatory, anti-cancer properties and regulatory functions of the hypothalamic–pituitary–adrenal axis^9–11,55,56^. However, CK being a minor ginsenoside is found in low amounts in the *Panax* rhizome and leaves^57^. The plant itself is a slow growing, perennial herb that is usually harvested only after 4–6 years and is challenged by sensitivity to various abiotic and biotic factors leading to low yield and variable ginsenoside content^58^. *Panax* spp. are grown in greenhouses and controlled environments to meet the demand for ginsenosides but are less preferred than those that are found naturally^59,60^. The high demands for the plant have led to widespread overexploitation of the plant in its natural habitat making it number negligible in the wild^61^.

Several methods for ginsenoside production are being sourced to meet this demand. Presently, the methods employed for CK production include microbial engineering, biotransformation, enzymatic and chemical processes^57,62–64^. The latter two processes are deemed expensive and low-yielding, bringing the focus on the former two with a preference for biotransformation. The biotransformation of major ginsenosides Rb1, Rd, Rc etc. to CK by human intestinal bacteria was well studied but the availability of CK was found to be highly influenced by the individual’s dietary and gut microflora^65^. Deglycosylation by endophytic glucosidases of *Arthrinium* sp. GE 17–18 from *P. ginseng* and *F. oxysporum* and *Coniochaeta* sp. isolated from *P. notoginseng* have also enhanced the yield of CK^41,63^. As new data sets of genomic studies on the biosynthetic gene clusters and metabolic pathways of the host plant and endophytes emerge, information on the possibility of microbial engineering, gene-knockout systems, and heterologous expression in yeasts is being explored^65–68^. Some endophytes can directly synthesize rare ginsenosides like Rg3 by *Chaetomium*^69^. The discovery of endophytes that could synthesize CK under in vitro was a possibility that researchers believed could enhance CK production. In this regard, our finding on the fungal endophytes of *P. sokpayensis* having CK production potential confirms that more diversity and screening studies on wild, endemic species of *Panax* can be employed for the discovery of endophytes that synthesize rare bioactive ginsenosides^70^. Further study on optimizing and improving the yield of CK by potential fungal endophytes is a prospect for future research work.

## Conclusion

In the present study, we report for the first time the fungal endophytes of *P. sokpayensis,* an endemic medicinal plant of Sikkim, India. Four fungal endophytes of *P. sokpayensis* exhibited the capability to produce rare ginsenoside CK under in vitro conditions, confirmed by using LC–ESI–MS/MS analysis which had never been discovered earlier. CK is of interest because of its high permeability and several therapeutic uses. The identification of a new source of the ginsenoside from the endophyte offers promise in exploiting the fungus as an alternative source for CK. Further studies are however required for the growth optimization as well as other culture conditions that would not only predispose the endophytes in stable production of the ginsenosides but also without any noticeable attenuation in the production over sub-culture generations. It could pave the way forward towards possible utilization of the endophytes to harness the medicinal property of the host plant without harvesting the rare plant from nature, which can restore the endemic plant in the wild and produce ginsenosides sustainably.

## Data Availability

The datasets generated and analyzed in the present study are available from the corresponding author upon reasonable written request.
